# Anti-Inflammatory and Anti-Allergic Effects of Saponarin and Its Impact on Signaling Pathways of RAW 264.7, RBL-2H3, and HaCaT Cells

**DOI:** 10.3390/ijms22168431

**Published:** 2021-08-05

**Authors:** Seon-Young Min, Che-Hwon Park, Hye-Won Yu, Young-Jin Park

**Affiliations:** Department of Medicinal Biosciences, Research Institute for Biomedical & Health Science, College of Biomedical and Health Science, Konkuk University, 268 Chungwon-daero, Chungju-si 27478, Korea; 124msy@kku.ac.kr (S.-Y.M.); chehwon9798@kku.ac.kr (C.-H.P.); ryu1hw@kku.ac.kr (H.-W.Y.)

**Keywords:** anti-inflammation, anti-allergy, atopic dermatitis, flavone, HaCaT, RBL-2H3, RAW264.7, saponarin

## Abstract

Saponarin{5-hydroxy-2-(4-hydroxyphenyl)-6-[3,4,5-trihydroxy-6-(hydroxymethyl)oxan-2-yl]-7-[3,4,5-trihydroxy-6-(hydroxymethyl)oxan-2-yl]oxychromen-4-one}, a flavone found in young green barley leaves, is known to possess antioxidant, antidiabetic, and hepatoprotective effects. In the present study, the anti-inflammatory, anti-allergic, and skin-protective effects of saponarin were investigated to evaluate its usefulness as a functional ingredient in cosmetics. In lipopolysaccharide-induced RAW264.7 (murine macrophage) cells, saponarin (80 μM) significantly inhibited cytokine expression, including tumor necrosis factor (TNF)-α, interleukin (IL)-1β, inducible nitric oxide synthase, and cyclooxygenase (COX)-2. Saponarin (80 μM) also inhibited the phosphorylation of extracellular signal-regulated kinase (ERK) and p38 involved in the mitogen-activated protein kinase signaling pathway in RAW264.7 cells. Saponarin (40 μM) significantly inhibited β-hexosaminidase degranulation as well as the phosphorylation of signaling effectors (Syk, phospholipase Cγ1, ERK, JNK, and p38) and the expression of inflammatory mediators (tumor necrosis factor [TNF]-α, IL-4, IL-5, IL-6, IL-13, COX-2, and FcεRIα/γ) in DNP-IgE- and DNP-BSA-stimulated RBL-2H3 (rat basophilic leukemia) cells. In addition, saponarin (100 μM) significantly inhibited the expression of macrophage-derived chemokine, thymus and activation-regulated chemokine, IL-33, thymic stromal lymphopoietin, and the phosphorylation of signaling molecules (ERK, p38 and signal transducer and activator of transcription 1 [STAT1]) in TNF-α- and interferon (IFN)-γ-stimulated HaCaT (human immortalized keratinocyte) cells. Saponarin (100 μM) also significantly induced the expression of hyaluronan synthase-3, aquaporin 3, and cathelicidin antimicrobial peptide (LL-37) in HaCaT cells, which play an important role as skin barriers. Saponarin remarkably inhibited the essential factors involved in the inflammatory and allergic responses of RAW264.7, RBL-2H3, and HaCaT cells, and induced the expression of factors that function as physical and chemical skin barriers in HaCaT cells. Therefore, saponarin could potentially be used to prevent and relieve immune-related skin diseases, including atopic dermatitis.

## 1. Introduction

Various natural products have fewer side effects compared to conventional synthetics and can effectively inhibit the excessive production of reactive oxygen species (ROS), preventing mutations and cytotoxicity. Therefore, there is currently much research underway aiming to identify novel physiologically active ingredients [1,2]. Many physiologically active substances have been discovered in plants and are widely used in functional foods, medicines, and cosmetics [3,4]. *Barley (Hordeum vulgare* L.) belongs to the family Poaceae (Gramineae), and in previous studies, we found that barley sprouts exhibit superior anti-allergic and anti-inflammatory activities [5]. In addition to various bioactive molecules, such as β-carotene, catechin, vitamin C, vitamin E, and quercetin, barley sprouts are reported to contain high levels of saponarin (isovitexin 7-O-glucoside or saponaretin-7-O-glucoside), according to the flavonoid database [6,7]. In addition, barley sprouts are rich in flavones, including isoschaftoside (apigenin 6-*C*-arabinoside-8-*C*-glucoside), isovitexin (apigenin 6-*C*-glucoside), schaftoside (apigenin 6-*C*-glucoside-8-*C*-arabinoside), 6-O-feruloyl, isovitexin 7-O-rutinoside, isovitexin 7-O-(6-O-feruloyl) glucoside-4′-O-glucoside, 6-O-sinapoylsaponarin (isovitexin 7-O-[6-O-sinapoyl] glucoside), and 6-O-sinapoylsaponarin (isovitexin 7-O-[6-O-sinapoyl] glucoside) [6,7].

Inflammation is associated with a variety of diseases. In particular, patients with atopic dermatitis (AD) have been reported to show increased expression of interleukin (IL)-4 and IL-13, the inflammatory mediators of T helper 2 cells (Th2), and also increased production of immunoglobulin E (IgE) [8]. Chemokines (thymus and activation-regulated chemokine; TARC/CCL17 and macrophage-derived chemokine; MDC/CCL22) and cytokines (IL-25, IL-33, and TSLP) are also essential inflammatory mediators in the development of AD, and their expression has been reported to increase in patients with AD [9,10,11]. It has been reported that mitogen-activated protein kinase (MAPK) and Janus tyrosine kinase/signal transducers and activators of transcription (JAK/STAT) signaling pathways are involved in the production of TARC and MDC in tumor necrosis factor (TNF)-α and interferon [IFN]-γ stimulated HaCaT (human immortalized keratinocyte) cells, and STAT1 induces the expression of various inflammatory genes such as TARC and MDC [12,13,14,15,16]. Chronic skin diseases in AD patients are caused by decreased expression of antimicrobial peptides (human β-defensin [HBD]-1, HBD-2, HBD-3, cathelicidin, secretory leukocyte proteinase inhibitor, dermcidin, and adrenomedullin) and abnormal skin components (involucrin, loricrin, filaggrin, hyaluronic acid, and aquaporins) [17,18,19,20,21,22,23].

The representative cell lines used for anti-inflammatory and anti-allergic activity studies are RBL-2H3 (rat basophilic leukemia) and RAW264.7 (murine macrophage). IgE binds to the high-affinity IgE receptor (FcεRI, a heterotetrameric receptor) of RBL-2H3 cells, promoting β-hexosaminidase degranulation and cytokine secretion, and triggering an allergic response. FcεRI induces phosphorylation of Lyn and Syk by IgE binding and continuously activates phospholipase C (PLC)-γ. In addition, MAPK signaling molecules are also activated by FcεRI-IgE crosslinking [24,25,26]. In RAW264.7 cells, inducible nitric oxide synthase (iNOS) is expressed by stimuli such as lipopolysaccharides (LPS), cytokines, and bacterial toxins, inducing nitric oxide (NO) production, which plays an important role in the immune response [27,28,29]. Cyclooxygenase (COX)-2, expressed primarily by inflammatory responses in RAW264.7 cells, triggers the production of prostaglandin E2 (PGE2), which is associated with fever and pain [30]. In addition, TNF-α plays an important role in inducing the inflammatory response and inducing the expression of pro-inflammatory cytokines, and IL-6 is a crucial inflammatory mediator secreted by LPS [31,32]. MAPK signaling molecules also play an important role in cell growth and differentiation, and induction of cytokine expression [33,34].

In order to utilize natural resources in various fields such as functional foods, medicines, and cosmetics, determining the physiological activities and mechanisms of the main ingredients contained in the resources is essential. Therefore, in this study, we investigated the anti-inflammatory and anti-allergic effects of saponarin, the main component of barley sprouts, as well as the effects on factors related to AD. We believe that the detailed mechanism of saponarin identified in this study provides a fundamental basis for various future uses of this flavonoid.

## 2. Results and Discussion

### 2.1. Cytotoxicity of Saponarin in RAW264.7, RBL-2H3, and HaCaT Cells

**Figure 1:**
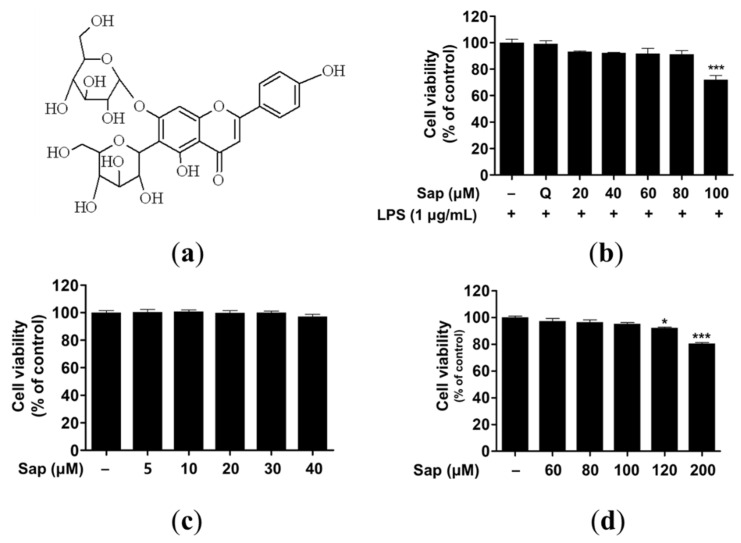
Molecular structure (**a**) and cytotoxic effects of saponarin on RAW264.7 (**b**), RBL-2H3 (**c**), and HaCaT cells (**d**). Cells were treated with various concentrations of saponarin (5–200 μM) for 24 h. Sap, saponarin; LPS, lipopolysaccharide; Q, quercetin (15 μM). The data were analyzed using one-way analysis of variance (ANOVA) followed by Tukey’s test. * *p* < 0.05, *** *p* < 0.001 versus cells without saponarin treatment.

Saponarin (C_27_H_30_O_15_) is a sugar-linked glycoside, a secondary plant metabolite, and is contained in sprout barley (Figure 1a and Figure S1). Despite health-promoting effects such as antioxidant and anti-inflammatory effects, several studies have reported adverse effects of flavonoids related to pro-oxidant activities [35,36,37]. Thus, we investigated the cytotoxicity of saponarin in RAW264.7, RBL-2H3, and HaCaT cells. As shown in Figure 1b–d, 100 and 120 μM saponarin showed significant cytotoxicity in RAW264.7 (72%, *p* < 0.001) (Figure 1b) and HaCaT (92.1%, *p* < 0.05) cells (Figure 1d), respectively. However, saponarin had no cytotoxic effect on RBL-2H3 cells at all concentrations (Figure 1c). Therefore, in the following experiments, RAW264.7, RBL-2H3, and HaCaT cells were treated with 80, 40, and 100 μM of saponarin, respectively.

### 2.2. Effects of Saponarin on NO Production and β-Hexosaminidase Release

**Figure 2:**
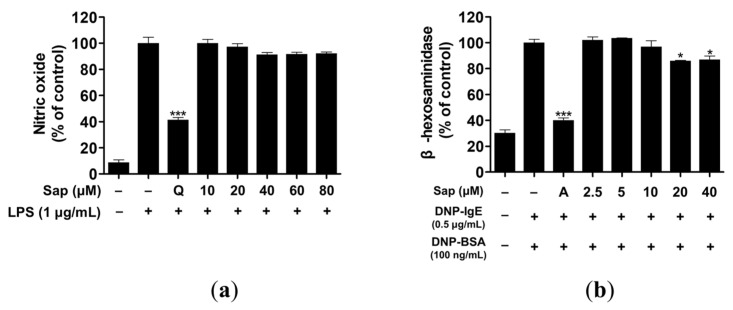
Effects of saponarin on nitric oxide production and β-hexosaminidase release of RAW264.7 (**a**) and RBL-2H3 cells (**b**), respectively. LPS- or IgE-induced cells were treated with various concentrations of saponarin (2.5~80 μM). Sap, saponarin; A, cyclosporine A (1 μg/mL); LPS, lipopolysaccharide; Q, quercetin (15 μM). The data were analyzed using one-way analysis of variance (ANOVA) followed by Tukey’s test. * *p* < 0.05, *** *p* < 0.001 versus LPS- or IgE-stimulated cells without saonarin treatment.

Macrophages produce inflammatory molecules, such as NO and pro-inflammatory cytokines, which play a crucial role in immune response and regulation [38]. However, overproduction of NO causes chronic inflammatory and autoimmune diseases [39]. We investigated the inhibitory activity of saponarin on NO production and found that saponarin did not inhibit NO production to a significant level in RAW264.7 cells (Figure 2a).

β-hexosaminidase is secreted by allergic reactions in the granules of mast cells and basophils. Therefore, assay of β-hexosaminidase activity is useful for discovering allergen inhibitors [40,41]. RBL-2H3 cells were treated with various concentrations (2.5, 5, 10, 20, 40 μM) of saponarin, and 20 and 40 μM saponarin significantly inhibited β-hexosaminidase activity in RBL-2H3 cells (Figure 2b).

### 2.3. Effects of Saponarin on Cytokines and the MAPK Signaling Pathway in RAW264.7 Cells

Since saponarin was not effective in inhibiting NO production, the effect on the ex-pression of cytokines, including TNF-α, IL-1β, IL-6, iNOS, and COX-2, which play an important role in the inflammatory response in RAW264.7 cells, was further analyzed. NO is involved in the inflammatory response along with inflammatory cytokines, including TNF-α, IL-1β, and IL-6, of which TNF-α can also be produced in activated macrophages, lymphocytes, mast cells, and endothelial cells [31,32,38]. IL-1β is a pro-inflammatory cytokine that is required for cell growth or maintenance of the low concentrations of homeostasis, but if overproduced, it can induce excessive inflammatory re-actions and exacerbate disease [42]. COX-1 is involved in platelet formation, gastrointestinal mucosa integrity, and maintenance of kidney function, but various anti-inflammatory agents act effectively by inhibiting COX-2 production or activity [43]. IL-6 is secreted from macrophages by LPS and plays an important role in the inflammatory response [32].

**Figure 3:**
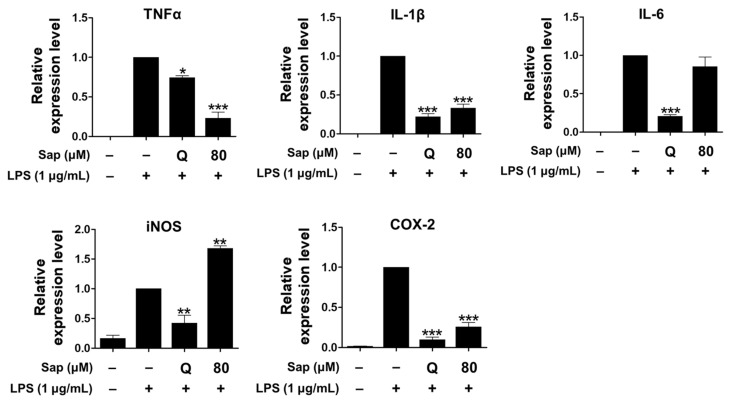
Effects of saponarin on the expression of cytokines in RAW264.7 cells. Cells were treated with saponarin (80 μM) for 24 h. Sap, saponarin; LPS, lipopolysaccharide; Q, quercetin (15 μM). The data were analyzed using one-way analysis of variance (ANOVA) followed by Tukey’s test. * *p* < 0.05, ** *p* < 0.01, *** *p* < 0.001 versus LPS-induced cells without saponarin treatment.

As shown in Figure 3, saponarin (80 μM) significantly inhibited the expression of TNF-α, IL-1β, and COX-2 in LPS-induced RAW264.7 cells. In addition, the expression of TNF-α was further reduced than quercetin (15 μM).

Saponarin did not inhibit the expression of iNOS, which is not present in cells but expressed by NF-κB, produces high levels of NO, and plays an important role in the overproduction of inflammatory molecules by LPS or cytokines in macrophages [44]. Thus, the increased NO productions is consequently due to the induction of iNOS expression.

**Figure 4:**
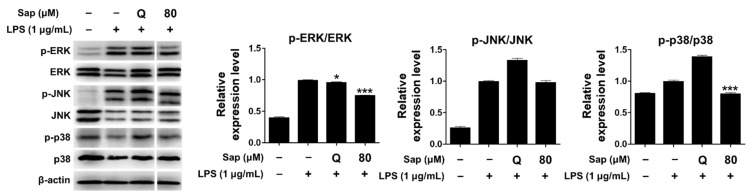
Effects of saponarin on the expression of mitogen-activated protein kinase (MAPK) signaling molecules in RAW264.7 cells. Cells were treated with saponarin (80 μM) for 24 h. Sap, saponarin; LPS, lipopolysaccharide; Q, quercetin (15 μM). The data were analyzed using one-way analysis of variance (ANOVA) followed by Tukey’s test. * *p* < 0.05, *** *p* < 0.001 versus LPS-induced cells without saponarin treatment.

Since saponarin significantly inhibited cytokines such as TNF-α, IL-1β, and COX-2, we further investigated the effect of saponarin (80 μM) on the MAPK signaling pathway. MAPKs (serine-threonine kinases), including ERK, p38, and JNK, are involved in cell growth and differentiation and play an important role in cellular responses to cytokines [33,34]. ERK is activated by various stimuli and phosphorylates various transcription factors, and p38 and JNK are activated by inflammatory cytokines during stress responses [45]. JNK is also involved in cytokine transcription and is activated by LPS stimulation [46,47]. As shown in Figure 4, it was found that the phosphorylation of ERK and p38 was suppressed in RAW264.7 cells (Figure 4). Thus, in the present study, we suggest that saponarin effectively suppresses the expression of inflammatory molecules including TNF-α, IL-1β, and COX-2 in RAW264.7 cells through inhibition of phosphorylation of MAPK signaling molecules, including ERK and p38.

### 2.4. Effects of Saponarin on Cytokines, MAPK, and Allergic Signaling Pathways in RBL-2H3 Cells 

**Figure 5:**
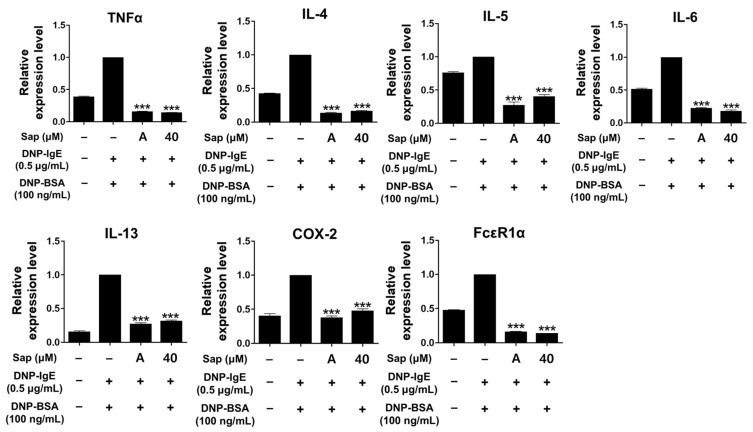
Effects of saponarin on the mRNA transcription of cytokines and FcεRIα in RBL-2H3 cells. Cells were treated with saponarin (40 μM), 0.5 μg/mL DNP-IgE, and 100 ng/mL DNP-BSA. Sap, saponarin; A, cyclosporine A (1 μg/mL). The data were analyzed using one-way analysis of variance (ANOVA) followed by Tukey’s test. *** *p* < 0.001 versus DNP-IgE- and DNP-BSA-treated cells without exposure to saponarin.

To investigate the inhibitory activity of saponarin on cytokines (TNF-α, IL-4, IL-5, IL-6, IL-13, and COX-2) and FcεRIα, RBL-2H3 cells were stimulated with DNP-IgE (0.5 μg/mL) and DNP-BSA (100 ng/mL). The inflammatory response of mast cells is regulated by cytokines such as IL-4, IL-5, IL-6, and IL-13, and TNF-α is the major cytokine of mast cells [48,49,50,51]. IL-4 promotes B cell differentiation and IgE synthesis, whereas IL-5 is involved in the differentiation of eosinophils and Th2 cells. IL-6 is expressed in the acute inflammatory response and plays a role in the exacerbation of Th2-mediated diseases such as asthma and allergic inflammation [49,50,51,52]. The expression of IgE is increased in AD, and IL-13 is an important regulator that induces IgE production along with IL-4 [53,54,55]. FcεRI in mast cells is activated by the binding of antigen-crosslinked IgE, which induces degranulation. Activated mast cells have been reported to increase the expression of the αβγ subunit of FcεRI, and activation of FcεRI induces an inflammatory response through subsequent reactions such as synthesis and secretion of various cytokines [56,57]. As shown in Figure 5, the mRNA expression of all molecules was significantly downregulated by saponarin treatment. Furthermore, the inhibitory activity of saponarin was similar to that of the positive control molecule (cyclosporine A, 1 μg/mL). In this study, saponarin (40 μM) significantly inhibited crucial inflammatory mediators, including TNF-α, IL-4, IL-5, IL-6, IL-13, COX-2, and FcεRIα in DNP-IgE- and DNP-BSA-induced RBL-2H3 cells. These results suggest that saponarin is effective against abnormal disorders involving excessive cytokine expression.

**Figure 6:**
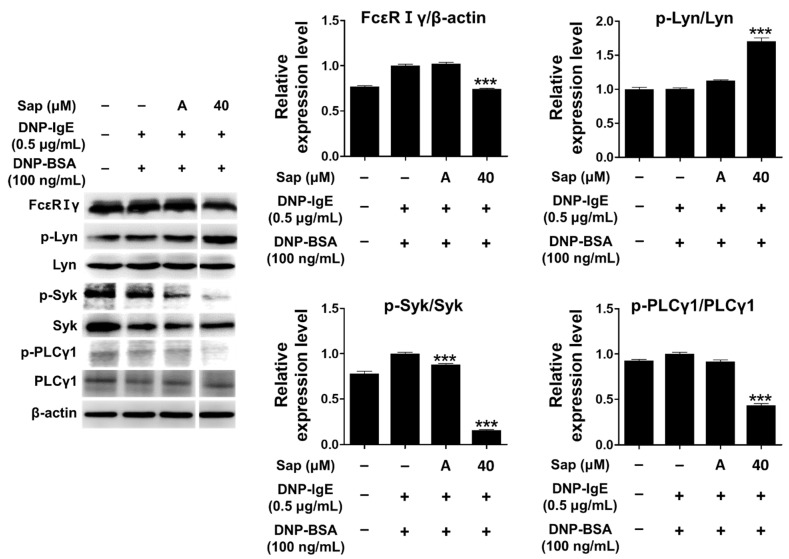
Effects of saponarin on IgE-activated signaling molecules in RBL-2H3 cells. Cells were treated with saponarin (40 μM), 0.5 μg/mL DNP-IgE, and 100 ng/mL DNP-BSA. Sap, saponarin; A, cyclosporine A (1 μg/mL). The data were analyzed using one-way analysis of variance (ANOVA) followed by Tukey’s test. *** *p* < 0.001 versus DNP-IgE- and DNP-BSA-treated cells without saponarin exposure.

The antigen-IgE complex activates Lyn (Src-family kinase) of the β-subunit due to the binding of the α-subunit of FcεRI in mast cells, and the γ-subunit is also activated [24,25,26,58]. Lyn kinase phosphorylates the tyrosine of the immunoreceptor-based activation motif (ITAM) in the β- and γ-subunits, providing a binding site for Syk kinase and inducing phosphorylation and activation of Syk kinase. Additionally, activation of Syk further activates signaling molecules such as phospholipase Cγ1 (PLCγ1) and MAPK, secreting other molecules that induce allergic responses [24,25,26,58,59,60]. Therefore, we investigated the inhibitory effect of saponarin on signal transduction in DNP-IgE- and DNP-BSA-induced RBL-2H3 cells, and saponarin (40 μM) significantly inhibited the phosphorylation of FcεRIγ-subunit and Syk as well as PLCγ1 (Figure 6). Although saponarin did not inhibit the phosphorylation of Lyn, these results indicate that saponarin can be used as a substance that effectively alleviates allergic responses by inhibiting the phosphorylation of FcεRIγ-subunit, Syk, and PLCγ1.

**Figure 7:**
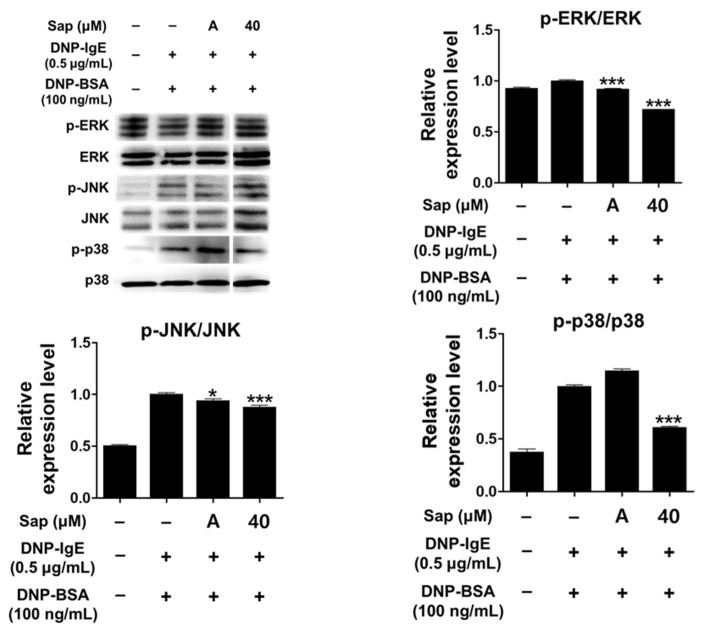
Effects of saponarin on mitogen-activated protein kinase (MAPK) in RBL-2H3 cells. Cells were treated with saponarin (40 μM), 0.5 μg/mL DNP-IgE, and 100 ng/mL DNP-BSA. Sap, saponarin; A, cyclosporine A (1 μg/mL). The data were analyzed using one-way analysis of variance (ANOVA) followed by Tukey’s test. * *p* < 0.05, *** *p* < 0.001 versus DNP-IgE- and DNP-BSA-treated cells without saponarin exposure.

Activation of RBL-2H3 cells by the antigen-IgE complex eventually activates MAPK signaling molecules. Activation of MAPK has been reported to induce the expression of various cytokines including TNF-α and IL-4 in mast cells, leading to inflammatory and allergic reactions [33,61,62,63]. We further evaluated whether saponarin inhibits phosphorylation of ERK, JNK, and p38. As shown in Figure 7, saponarin (40 μM) significantly reduced the phosphorylation of ERK, JNK, and p38 in DNP-IgE- and DNP-BSA-induced RBL-2H3 cells. Therefore, inhibition of cytokines induced by DNP-IgE and DNP-BSA in RBL-2H3 cells (Figure 5) is consequently due to inhibition of phosphorylation of MAPK signaling molecules, suggesting that saponarin is a substance that effectively controls inflammatory and allergic responses.

### 2.5. Effects of Saponarin on Cytokines and the MAPK and STAT1 Pathways in HaCaT Cells

TARC (CCL17) is produced in tissue cells and macrophages, such as skin endothelial cells, epithelial cells, keratinocytes, and fibroblasts through the activation of MAPK and nuclear factor kappa B (NF-κB) pathways by the interaction between IL-4 and transforming growth factor-β [9]. MDC (CCL22) is produced in dendritic cells, B cells, macrophages, keratinocytes, and epithelial cells [10]. According to a recent study, TARC and MDC measured in cord blood were significantly increased in children with allergic symptoms before 6 years of age compared to normal children [11]. In addition, it was reported that CCR4+ Th2 cells infiltrate the skin during the acute exacerbation of AD (AD), resulting in increased expression of TARC and MDC, but symptoms improved after steroid treatment, and decreased expression of TARC and MDC [10]. Th2 cytokines such as IL-4, IL-5, and IL-13 levels vary greatly depending on the sample type and sampling time, while Th2-related chemokines, such as TARC and MDC, are highly reproducible and stably measured in serum, plasma, sputum, and bronchial alveolar fluid. As a result, they are attracting attention as biomarkers reflecting the diagnosis and severity of allergic diseases such as asthma and AD, and are being actively studied as targets of novel immunomodulatory treatments [64,65]. Interleukin-33 (IL-33) is highly secreted in patients with AD, and transgenic mice secreting approximately 10 times more IL-33 than wild-type mice showed symptoms of AD, such as itching and skin thickening [66]. IL-33 is a nuclear protein released from damaged epithelial cells and acts as a signaling molecule, while IL-25 is constitutively expressed in the cellular compartment of epithelial cells and released by allergen proteases [67,68]. Thymic stromal lymphopoietin (TSLP), which is present in high concentrations in keratinocytes of AD patients, is a protein that acts preferentially in abnormal signaling [69]. In particular, it is reported that TSLP stimulates CD11c+ dendritic cells via allergens and increases TARC and MDC, which is closely related to the occurrence of AD [70].

**Figure 8:**
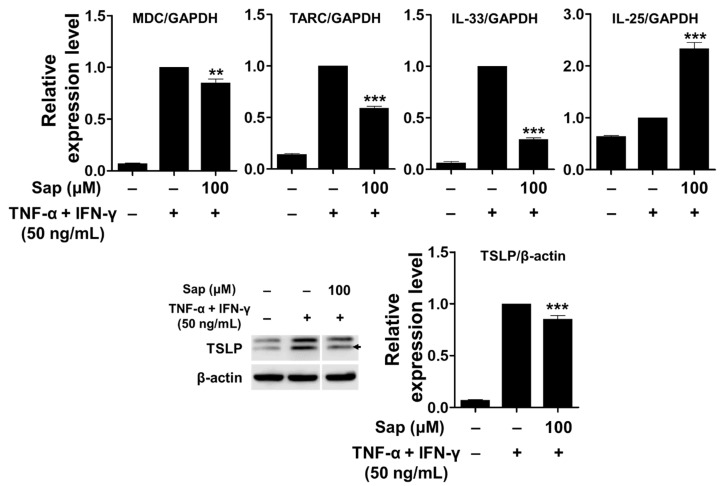
Effects of saponarin on chemokines and cytokines in HaCaT cells. Cells (3 × 10^5^ cells/well) were treated with saponarin (100 μM) for 18 h, and then with a mixture of recombinant human TNF-α and IFN-γ (each 50 ng/mL) for 6 h. Sap, saponarin. The data were analyzed using one-way analysis of variance (ANOVA) followed by Tukey’s test. ** *p* < 0.01, *** *p* < 0.001 versus TNF-α- and IFN-γ-treated cells without saponarin exposure.

To investigate the inhibitory effect of saponarin on chemokines (MDC and TARC) and cytokines (IL-25, IL-33, and TSLP), saponarin-pretreated HaCaT cells were stimulated with TNF-α and IFN-γ. As shown in Figure 8, mRNA expression of MDC, TARC, and IL-33 was upregulated by TNF-α and IFN-γ stimulation, and these increases were significantly inhibited by saponarin pretreatment (100 μM). In addition, the expression of TSLP protein was also significantly decreased by saponarin. These results suggest that saponarin can modulate the expression of Th2 chemokines (MDC and TARC) and cytokines (IL-33 and TSLP) in TNF-α- and IFN-γ-stimulated HaCaT cells. However, it was shown that pretreatment with saponarin increased the mRNA expression of IL-25. Therefore, it was found that saponarin only inhibits the expression of specific chemokines and cytokines in the HaCaT cells.

**Figure 9:**
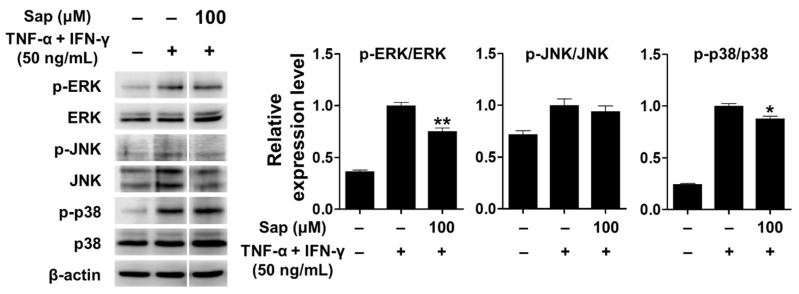
Effects of saponarin on MAPK (mitogen-activated protein kinase) signaling molecules in HaCaT cells. Cells (5 × 10^5^ cells/well) were treated with saponarin (100 μM) for 1 h, and then with a mixture of recombinant human TNF-α and IFN-γ (each 50 ng/mL) for 15 min. Sap, saponarin. The data were analyzed using one-way analysis of variance (ANOVA) followed by Tukey’s test. * *p* < 0.05, ** *p* < 0.01 versus TNF-α- and IFN-γ-treated cells without saponarin exposure.

TNF-α, a cytokine important in the inflammatory response, activates various signaling pathways [71]. In addition, previous studies reported that p38 of the MAPK signaling pathway plays an important role in the expression of TARC and MDC in HaCaT cells stimulated by TNF-α and IFN-γ [12,13,14,15]. Therefore, we investigated whether saponarin inhibits the activation of MAPK signaling molecules, including ERK, JNK, and p38, in TNF-α- and IFN-γ-stimulated HaCaT cells. As shown in Figure 9, MAPK signaling molecules were activated by TNF-α and IFN-γ stimulation in HaCaT cells. Saponarin (100 μM) was rather found to decrease the phosphorylation of ERK and p38 in TNF-α- and IFN-γ-stimulated HaCaT cells. These results suggest that saponarin does inhibit MDC and TARC in HaCaT cells stimulated by TNF-α and IFN-γ through inhibiting the activation of MAPK signaling molecules.

**Figure 10:**
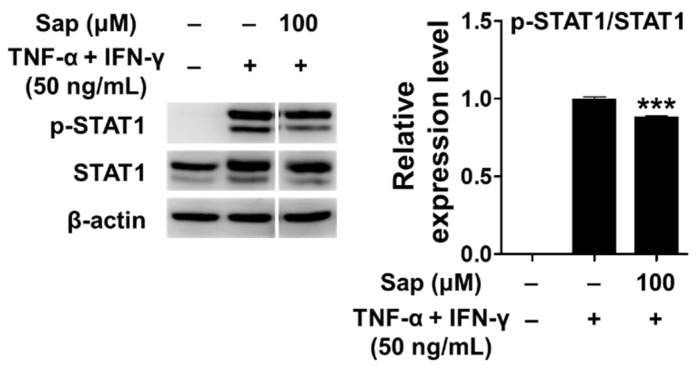
Effects of saponarin on signal transducer and activator of transcription 1 (STAT1) signaling molecules in HaCaT cells. Cells were treated with saponarin (100 μM), 50 ng/mL TNF-α, and 50 ng/mL IFN-γ. Sap, saponarin. The data were analyzed using one-way analysis of variance (ANOVA) followed by Tukey’s test. *** *p* < 0.001 versus TNF-α- and IFN-γ-treated cells without saponarin exposure.

Previous studies reported that the JAK/STAT (Janus tyrosine kinase/signal transducers and activators of transcription) signaling pathway, along with MAPK, is also involved in the production of TARC and MDC in TNF-α- and IFN-γ-stimulated HaCaT cells [12,16]. Receptor binding of IFN-γ activates the JAK/STAT signaling pathway, and Janus tyrosine kinase (JAK) regulates tyrosine phosphorylation of STAT proteins [72]. STAT1 is an important transcription factor activated by stimuli such as TNF-α and IFN-γ, and translocates to the nucleus to induce the expression of various inflammatory genes [12,16]. Con-sequently, we investigated the effect of saponarin on the phosphorylation of STAT1 protein in TNF-α- and IFN-γ-stimulated HaCaT cells. As shown in Figure 10, STAT1 was phosphorylated by TNF-α and IFN-γ stimulation, but phosphorylation of STAT1 protein was significantly reduced by pretreatment with saponarin. Crosstalk refers to a change in which the degree of activation of an intracellular delivery substance for one stimulus in-creases or decreases by the intracellular delivery process of another stimulus. It has been found that phosphorylation of S727 is essential for the transcriptional activity of STAT1. Since S727 is located at the potential active site (Pro-Met-Ser-Pro; PMSP) of MAPK, it has been reported that crosstalk between the Jak/STAT pathway and the MAPK pathway is mediated by the STAT molecule [73,74]. In this study, there was a crosstalk effect of saponarin on the MAPK and JAK/STAT pathways, and the expression of MDC and TARC was suppressed by the inhibition of STAT1 activity in the JAK/STAT pathway.

### 2.6. Effects of Saponarin on Other Molecules in HaCaT Cells

**Figure 11:**
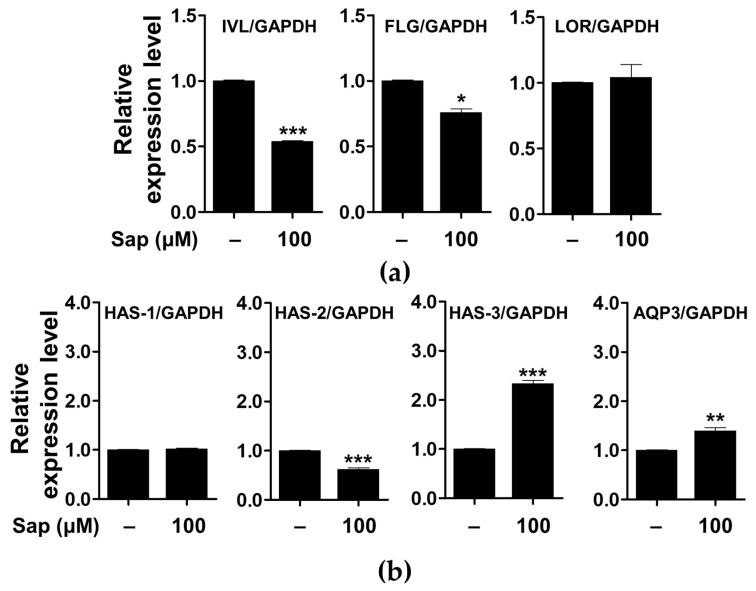
Effects of saponarin on the expression of the genes related to the physical barrier functions (**a**) and skin hydration (**b**) of HaCaT cells. Cells were treated with saponarin (100 μM) for 24 h. Sap, saponarin; IVL, involucrin; FLG, filaggrin; LOR, loricrin; HAS-1, hyalu-ronic acid synthesis 1; HAS-2, hyaluronic acid synthesis 2; HAS-3, hyaluronic acid synthesis 3; AQP3, aquaporin 3. The data were analyzed using the Student’s *t*-test. * *p* < 0.05, ** *p* < 0.01, *** *p* < 0.001 versus cells without saponarin treatment.

In addition to the evaluation of inflammatory mediators and signaling molecules involved in AD, we further investigated the effect of saponarin on factors related to the physical structure of HaCaT cells. As shown in Figure 11, saponarin (100 μM) treatment significantly increased the expression of the gene encoding HAS-3, which is involved in hyaluronic acid biosynthesis, and AQP3 is involved in maintaining moisture in HaCaT cells. Filaggrin, involucrin, and loricrin are major constituent proteins of the skin, and in particular, filaggrin and involucrin are downregulated by damage to the skin barrier. In addition, a relationship between AD, asthma, and allergic rhinitis and mutations in the filaggrin gene has been reported [17,75,76]. AQP is a protein involved in transporting water and glycerol into keratinocytes and has been reported to play an important role in maintaining skin moisture [77,78,79,80,81,82,83,84]. In addition, it has been reported that the reduction of HA, a major component of the extracellular matrix of the skin, is associated with the occurrence of psoriasis and skin inflammation along with wrinkle formation and elasticity reduction [85]. HA is synthesized by the HAS gene (HAS-1, HAS-2, HAS-3) in keratinocytes, and a decrease in HAS gene expression has been reported to induce defects in the moisturizing barrier, and is used as an indicator to evaluate the skin moisturizing effect [85,86,87]. Saponarin did not increase the expression of filaggrin, involucrin, loricrin, HAS-1, and HAS-2, but suggests that it will be effective against abnormal skin disorders through HAS-3 and AQP-3 induction as well as inhibition of inflammatory mediators.

**Figure 12:**
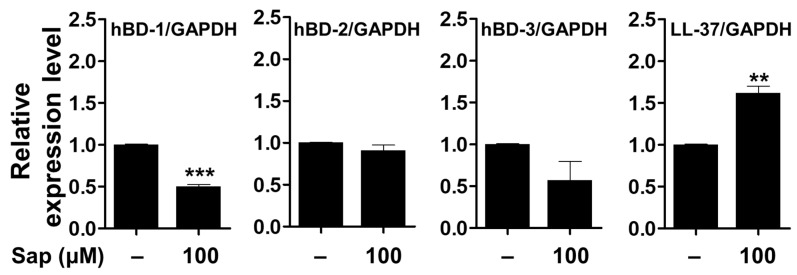
Effects of saponarin on the expression of antimicrobial peptides in HaCaT cells. Cells were treated with saponarin (100 μM) for 24 h. Sap, saponarin; hBD-1, beta-Defensin-1; hBD-2, beta-Defensin-2; hBD-3, beta-Defensin-3; LL-37, cathelici-din. The data were analyzed using the Student’s *t*-test. ** *p* < 0.01, *** *p* < 0.001 versus cells without saponarin treatment.

It has been reported that skin barrier damage caused by chronic skin diseases, such as AD and psoriasis, promotes the penetration of allergens and is associated with decreased expression of antimicrobial peptides [88,89,90]. Antimicrobial peptides such as HBD and cathelicidin (LL-37), produced in keratinocytes of the epidermis, act as chemical barriers against invading pathogens and exert antibacterial activity [17,18,19,91]. Among the HBDs, which are cationic antibacterial peptides mainly expressed in human skin, HBD-1 is constitutively expressed in the epithelium and sweat glands under normal conditions. HBD-2 and HBD-3 are expressed by keratinocyte differentiation, bacterial infection, and cytokine stimulation (IL-1β and TNF-α) [92,93,94]. In addition, LL-37, which is also involved in maintaining the function of the skin barrier, exerts antimicrobial activity against fungi and viruses as well as bacteria [20]. Antimicrobial peptides, which are rapidly expressed by external infectors, are an important component of innate immunity to protect the skin, and the reduction of antimicrobial peptides decreases immunity against pathogens, leading to psoriasis and inflammatory skin diseases [88,90,95]. Thus, we investigated the effect of saponarin on the expression of antimicrobial peptides in HaCaT cells. As shown in Figure 12, the mRNA expression of the genes encoding HBD-1, HBD-2, and HDB-3 proteins was decreased, although the LL-37 gene was significantly increased by saponarin treatment. Although the expression of HBD antimicrobial peptides did not increase, saponarin significantly increased the expression of LL-37 antimicrobial peptide. These results suggest that saponarin will be useful in mitigating inflammatory skin diseases such as psoriasis, acne, and AD by activating innate immunity and protecting the skin from invasion by external microbes due to the induction of LL-37 expression.

## 3. Materials and Methods

### 3.1. Reagents

Saponarin (purity > 98%) was obtained from Extrasynthese (Genay, France). Dulbecco’s modified Eagle’s medium (DMEM), antibiotics (penicillin and streptomycin), and trypsin-ethylenediaminetetraacetic acid (EDTA) were obtained from Gibco BRL (Grand Island, NY, USA). Fetal bovine serum (FBS) was purchased from Biowest (Kansas City, MO, USA), and lipopolysaccharide (LPS), monoclonal anti-DNP-IgE, 3-(4,5-dimethylthiazol-2-yl)-2,5-diphenyltetrazolium bromide (MTT), and 4-nitrophenyl n-acetyl-b-d-glucosaminide (p-NAG) were obtained from Sigma-Aldrich (St. Louis, MO, USA). DNP-BSA was procured from Invitrogen (Carlsbad, CA, USA). Recombinant human TNF-α and IFN-γ were purchased from Peprotech (Rocky Hill, NJ, USA). Primary anti-bodies against ERK, JNK, p38, Lyn, Syk, PLCγ, STAT1, p-ERK, p-JNK, p-p38, p-STAT1, p-Lyn, p-Syk, p-PLCγ, and β-actin were purchased from Cell Signaling Technology (Danvers, MA, USA). Primary antibody against TSLP was purchased from ABclonal (Woburn, MA, USA). Primary antibodies against FcεRIγ were purchased from LSBio (Seattle, WA, USA). The SensiFAST SYBR No-ROX kit mix was purchased from Biolines (Seoul, Korea). RAW264.7 (ATCC^®^ TIB-71) and RBL-2H3 (ATCC^®^ CRL-2256) cells were purchased from the American Type Culture Collection (ATCC). HaCaT cells were obtained from Byoung-Woo Lim of Konkuk University, Korea.

### 3.2. Cell Culture and Cell Viability Assay

Cells (RBL-2H3, RAW264.7, and HaCaT) were maintained in DMEM (10% FBS and 1% antibiotics [penicillin and streptomycin]) and incubated at 37 °C in a 5% CO_2_ humidified incubator. Cell viability was assessed using the MTT assay [96]. Briefly, RAW264.7 cells were treated with saponarin (20, 40, 60, 80, and 100 μM) in the presence or absence of LPS (1 μg/mL). RBL-2H3 and HaCaT cells were treated with saponarin (for RBL-2H3 cells 5, 10, 20, 30, and 40 μM; HaCaT cells, 60, 80, 100, 120, and 200 μM). After incubation for 24 h, MTT solution (0.5 mg/mL) was added to each well and the supernatants were discarded. Formazan crystals were dissolved in dimethyl sulfoxide (DMSO) and optical absorbance (at 570 nm) was measured using a microplate reader (TECAN, Männedorf, Switzerland).

### 3.3. NO and β-Hexosaminidase Release Assay

RAW264.7 cells (5 × 104 cells/well, 24-well plates) were seeded and incubated for 24 h. The cells were stimulated with LPS (1 μg/mL) and treated with various concentrations of saponarin (20, 40, 60, and 80 μM) for 24 h. After incubation, the supernatant was mixed with Griess reagent for 10 min, and the optical absorbance was measured at 530 nm using a microplate reader. The amount of NO produced was calculated using a sodium nitrite (NaNO_2_) standard curve.

RBL-2H3 cells (2 × 105 cells/well, 24-well plates) were seeded and treated with 0.5 μg/mL DNP-IgE for 24 h. After incubation, the cells were washed with Tyrode buffer (2.5 mM calcium chloride [CaCl_2_], 119 mM sodium chloride [NaCl], 1.19 mM magnesium sulfate [MgSO_4_], 5 mM potassium chloride [KCl], 5 mM glucose, 10 mM HEPES, and 1 mg/mL BSA, pH 7.3) and incubated with various concentrations of saponarin (5, 10, 20, and 40 μM) for 20 min, then with 100 ng/mL DNP-BSA for 1 h. The supernatants (50 μL) were incubated with a substrate buffer (3.3 mM p-nitrophenyl-N-acetyl-β-D-glucosaminide, pH 4.5) at 37 °C for 1 h. The reaction was terminated using a stop solution (0.1 M sodium bicarbonate [NaHCO_3_] and sodium carbonate [Na_2_CO_3_], pH 10.2), and the optical absorbance was measured at 407 nm using a microplate reader.

### 3.4. Real-Time Quantitative PCR

Total RNA was isolated using TRIzol Reagent (Thermo Scientific, Seoul, Korea) ac-cording to the manufacturer’s instructions. cDNA was synthesized using M-MLV RTase (Bioneer, Daejeon, Korea) according to the manufacturer’s instructions. Real-time quantitative PCR was performed on a Rotor-Gene 6000 (Qiagen, Seoul, Korea) using the Sensi-Fast SYBR No-ROX kit and primers (Supplementary Table S1) according to the manufacturer’s instructions. Relative quantification of the target gene was calculated using the ^Δ^^Δ^Ct method.

### 3.5. Western Blot Analysis

Cells were lysed with radioimmunoprecipitation (RIPA) buffer (Thermo Scientific, Seoul, Korea) containing a phosphatase inhibitor cocktail and protease inhibitor cocktail (GenDEPOT, Seoul, Korea). Total protein concentration was measured using Bradford re-agent (Bio-Rad, Seoul, Korea) at 595 nm. Proteins (8–20 μg) were electrophoresed using sodium dodecyl sulfate-polyacrylamide gel electrophoresis (SDS-PAGE) and transferred onto a polyvinylidene fluoride (PVDF) membrane (Merck Millipore, Seoul, Korea). The membrane was blocked with 3–5% bovine serum albumin (BSA) (GenDEPOT, Seoul, Korea) and reacted with 1:1000 diluted primary antibodies (ERK, JNK, p38, Lyn, Syk, PLCγ, FcεRIγ, STAT1, p-ERK, p-JNK, p-p38, p-Lyn, p-Syk, p-PLCγ, p-STAT1, TSLP, and β-actin) at 4 °C for 16 h. Protein signals were visualized using horseradish peroxidase (HRP)-conjugated secondary antibodies (Santa Cruz Biotech, Dallas, TX, USA) and the EZ-western Lumi Femto^TM^ Kit (Dogen, Seoul, Korea), and then scanned using a C-Digit blot scanner (LI-COR Biosciences, Lincoln, NE, USA). Relative expression levels were quantified using ImageJ software (National Institutes of Health, Bethesda, MD, USA).

### 3.6. Statistical Analysis

All data are expressed as mean ± standard deviation (SD) of three independent experiments. Statistical significance was evaluated using the Student’s *t*-test and one-way analysis of variance (ANOVA), followed by Tukey’s test using Prism software (GraphPad Software Inc., La Jolla, CA, USA).

## 4. Conclusions

In the present study, we evaluated the effects of saponarin on the factors associated with inflammation, allergy, and AD in RAW264.7, RBL-2H3, and HaCaT cells. Saponarin (80 μM) inhibited cytokine expression (IL-1β, IL6, iNOS, and COX-2) as well as the phosphorylation of ERK and p38 in the MAPK pathway in RAW264.7 cells. Saponarin (40 μM) also inhibited the phosphorylation of signaling molecules (Syk, PLCγ1, ERK, JNK, and p38) and the expression of inflammatory mediators (TNF-α, IL-4, IL-5, IL-6, IL-13, COX-2, and FcεRIα/γ) as well as β-hexosaminidase degranulation, which are essential for allergic responses in RBL-2H3 cells. In addition, saponarin (100 μM) significantly inhibited the expression of chemokines (MDC, TARC) and cytokines (IL-33 and TSLP) as well as the phosphorylation of signaling effectors (ERK, p38 and STAT1) in HaCaT cells stimulated with TNF-α and IFN-γ, which are essential for the development of AD. Moreover, saponarin (100 μM) significantly increased the expression of HAS-3, AQP3, and antimicrobial peptides (LL-37), which play an important role in the physical and chemical barrier of the skin. Although further research is needed, our results suggest that saponarin is a valuable candidate for alleviating inflammation, allergies, and AD.

## Supplementary Materials

The following are available online at https://www.mdpi.com/article/10.3390/ijms22168431/s1.
