# Supplementary material for: Anti-Inflammatory and Anti-Allergic Effects of Saponarin and Its Impact on Signaling Pathways of RAW 264.7, RBL-2H3, and HaCaT Cells
Source: Int J Mol Sci. 2021 Aug 5;22(16):8431. doi: 10.3390/ijms22168431 (PMC8395081; doi:10.3390/ijms22168431)
Supplement: Supplementary file 1 [file ijms-22-08431-s001.zip › Supplementary Figure S1.pptx]

## Slide 1
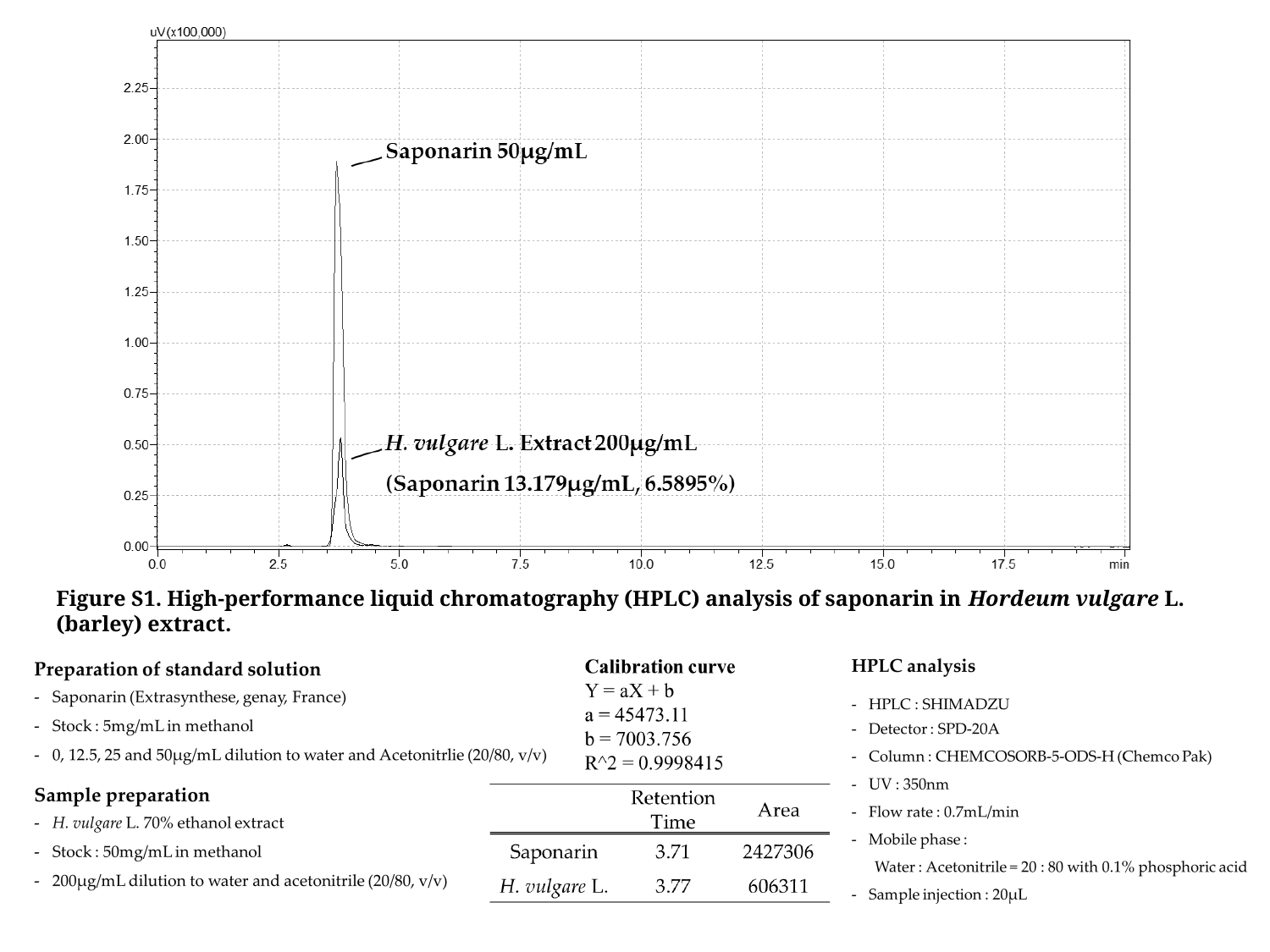

Figure S1. High-performance liquid chromatography (HPLC) analysis of saponarin in Hordeum vulgare L. (barley) extract.
